# Methods for the separation of hydraulic retention time and solids retention time in the application of photosynthetic microorganisms in photobioreactors: a review

**DOI:** 10.1007/s11274-024-03909-z

**Published:** 2024-02-17

**Authors:** Grant Keet, J. P. Du Toit, Robert William McClelland Pott

**Affiliations:** 1 Department of Chemical Engineering, Stellenbosch University, Stellenbosch, South Africa; 2 Watchmaker Genomics, Cape Town, South Africa

**Keywords:** Photosynthetic microorganisms, Immobilisation, Hydraulic retention time, Solid retention time, Biotechnical applications

## Abstract

Photosynthetic microorganisms have a wide range of biotechnical applications, through the application of their versatile metabolisms. However, their use in industry has been extremely limited to date, partially because of the additional complexities associated with their cultivation in comparison to other organisms. Strategies and developments in photobioreactors (PBRs) designed for their culture and applications are needed to drive the field forward. One particular area which bears examination is the use of strategies to separate solid- and hydraulic-residence times (SRT and HRT), to facilitate flow-through systems and continuous processing. The aim of this review is to discuss the various types of PBRs and methods which are currently demonstrated in the literature and industry, with a focus on the separation of HRT and SRT. The use of an efficient method of biomass retention in a PBR may be advantageous as it unlocks the option for continuous operation, which may improve efficiency, and improve economic feasibility of large-scale implementation of photosynthetic biocatalysts, especially where biomass is not the primary product. Due to the underexplored nature of the separation of HRT and SRT in reactors using photosynthetic microorganisms, limited literature is available regarding their performance, efficiencies, and potential issues. This review first introduces an overview into photosynthetic microorganisms cultivated and commonly exploited for use in biotechnological applications, with reference to bioreactor considerations specific to each organism. Following this, the existing technologies used for the separation of HRT and SRT in PBRs are explored. The respective advantages and disadvantages are discussed for each PBR design, which may inform an interested bioprocess engineer.

## Introduction

Photosynthetic microorganisms have a wide variety of biotechnical applications, from the wastewater treatment industry or bioremediation of complex waste streams (Harayama et al. 1999; Gao et al. 2017; Narayanan and Narayan 2019; Li et al. 2021), to commodity chemical production such as microbial oils (Economou et al. 2015; Maltsev et al. 2021), to speciality chemicals such as pharmaceuticals (Ho et al. 2021), or a future source of green energy and biohydrogen production (Vincenzini et al. 1982a; Ghirardi et al. 2009). However, to date, the abilities and application of only a few of these microorganisms have been investigated in the literature or applied in industry.

Due to their wide range of metabolic capabilities, photosynthetic microorganisms can be viewed as biological chassis organisms available for exploitation to produce valuable materials or for the bioremediation of challenging waste streams. Low value waste streams containing an organic source of carbon can often be employed as feedstocks to these photofermentative bioprocesses, with illumination provided artificially or exploiting natural sunlight as the primary source.

Currently there are there are a variety of PBRs employed in research and, less frequently, in industry. The majority of PBRs are designed for batch culture of the organism in question, which works reasonably well if the product of interest is the biomass itself. However, there are several use cases for photosynthetic organisms where the system would benefit from the biomass remaining in the PBR as media flows through the PBR. A significant limitation for this sort of continuous operation is cell washout, which occurs when the dilution rate is higher than the biomass growth rate (Maier and Pepper 2015). To simplify the application of PBRs in industry, methods need to be developed for retaining biomass while still allowing for liquid medium to pass through in a continuous operating fashion.

Biomass retention is achieved by designing a system that decouples solids retention time (SRT) and hydraulic retention time (HRT) of the microorganisms and media in the bioprocess (Marbelia et al. 2014). The SRT of a system is the average amount of time a solid particle, in this case a photosynthetic microorganism cell, remains in the PBR. Similarly, the HRT of a system is the average amount of time that a soluble compound or volume of liquid, in this case whichever medium is used, remains in the PBR.

Although a variety of PBRs exist in industry or have been explored in literature for several biotechnical applications, the concept of the separation of HRT and SRT in PBRs for photosynthetic microorganisms is comparatively underexplored and there is limited literature focussed on the topic. The aim of this review is therefore to examine the current literature that is available regarding PBRs demonstrated in literature and industry, or novel designs, focusing on the separation of HRT and SRT within PBRs. This is done first through an overview into the biology and uses of photosynthetic microorganisms commonly cultivated (which gives insight into how each class of organism might be applied in different PBR systems), followed by a discussion of existing technologies and PBR designs which promote the separation of HRT and SRT. This investigation into PBR designs and technologies includes the suitability of specific photosynthetic microorganisms as well as advantages and disadvantages of each design with respect to the photosynthetic microorganism and overall bioprocess. This review article will assist and inform researchers investigating photosynthetic organisms, and move towards industrial application.

## Types of photosynthetic microorganisms cultivated

Photosynthetic microorganisms which are commonly cultivated in industry for biotechnical applications or research purposes include green algae, cyanobacteria, purple non-sulfur bacteria (PNSB), purple sulfur bacteria (PSB), green non-sulfur bacteria (GNSB) and green sulfur bacteria (GSB). Although only a few of these microorganisms have been widely investigated, there are many photosynthetic microorganisms which have the potential to serve a purpose in industry. Despite the wide range of photosynthetic microorganisms available for biological exploitation, there are comparatively few examples of the commercial application of PSB, GNSB and GSB in literature, especially cases exhibiting the separation of HRT and SRT. Each of these microorganisms can be applied to different applications depending on the desired products to produce or the compounds to remediate, taking into consideration the environmental conditions, substrates available and the suitability of the photosynthetic microorganism thereof.

Green algae are a group of chlorophyll containing photosynthetic microorganisms belonging to the Chlorophyta group. Various species of green algae boast a wide variety of research and industrial applications, of which *Chlorella vulgaris* has received a significant portion of the attention, due to its robust metabolism and efficient photosynthetic ability (Liu and Chen 2016). Similarly to green algae, cyanobacteria have a wide variety of species which have been applied and have the potential to be used in biotechnical applications. Species of cyanobacteria including *Arthrospira platensis, Synechococcus,* and *Synechocystis* have been subject to research and cultivated at commercial scales for use in the wastewater treatment industry (Olguín and Sánchez-Galván 2011; Cepoi and Zinicovscaia 2020; Wan et al. 2021) or to produce human or animal nutritional supplements (Matos 2019; Wan et al. 2021), pharmaceuticals (Matos 2019; Wan et al. 2021), bio-lipids (Neag et al. 2019; Wan et al. 2021), hydrogen or biogas (Bohutskyi and Bouwer 2012; Wan et al. 2021).

Another relatively widely investigated group of phototrophic microorganisms are the purple non-sulfur bacteria, an extremely metabolically versatile group of microorganisms. The most commonly observed and studied species in literature under this group include *Rhodobacter sphaeroides, Rhodobacter capsulatus, Rhodospirillum rubrum* and *Rhodopseudomonas palustris* (Madigan and Jung 2009). These microorganisms have been cultivated for use in wastewater treatment or to biologically produce high purity hydrogen gas. The performance of these microorganisms for hydrogen production has been extensively investigated although little research has been conducted to combine both wastewater treatment and hydrogen production in one single PBR. The common goal for the application of these microorganisms in industry is generally to produce a valuable product (sometimes this product is biomass) or to treat wastewater streams containing organic or inorganic contaminants. The main pitfall resulting in the current economic unfeasibility for the industrial implementation of PBRs are the low conversion efficiencies generally obtained, which can be primarily attributed to inefficient light energy utilization and comparatively slow cell growth rates (Tian et al. 2010). Poor volumetric growth rates might be improved by retaining biomass in the reactor system while light conversion efficiency is largely affected by the PBR structure and design (Swineharf et al. 1962; Torzillo et al. 2022). Thus, the main motivation behind the need to separate HRT and SRT is the need to retain biomass in the PBR system, opening the possibility of efficient continuous processing.

A noticeable gap in the literature was observed for many of these photosynthetic microorganisms that have the potential to be applied to the biotechnical industry but currently face the main challenges associated with cell washout and the effects of mutual shading. Cell washout occurs when the medium flow rate is higher than the rate at which the microorganisms can reproduce to maintain a population within the bioreactor (Foutch and Johannes 2003). Mutual shading refers to the process whereby photosynthetic microorganisms absorb light for their own use, shading microorganisms further away from the light source—this effect becomes more apparent at higher biomass concentrations, and can result in significant portions of the reactor volume being completely shaded. These challenges limited their large scale and commercial application, even though their employment can be environmentally and economically beneficial. Through the separation of the HRT and SRT in PBRs using various methods of entrapment or separation of the microorganism, these challenges might be mitigated or minimized, driving forward the large-scale commercial application of photosynthetic biotechnology.

## Separation of HRT and SRT in photobioreactors

### Introduction to the separation of hydraulic and solids retention in photobioreactors

A PBR is a specific type of (usually) enclosed reactor with characteristics suitable for growing and controlling phototrophic bacteria, cyanobacteria, or algae for the biological production of valuable products or the bioremediation of waste (Znad 2020). PBRs come in various forms, equipped with mechanisms to provide adequate lighting to the microorganism, agitation of the medium and culture, removal of oxygen or unwanted by-products and recovery of the desired product. Most of these PBRs currently operate in batch mode where the biomass is cultivated followed by a separation stage or in a continuous flow mode, accounting for biomass exiting the system with the effluent. Batch processing conditions are generally an undesirable method of commercial operation in the bioprocessing industry due to the large down time associated with cleaning, sterilization and startup, leading to low overall productivities (Srivastava and Gupta 2011).

**Fig. 1:**
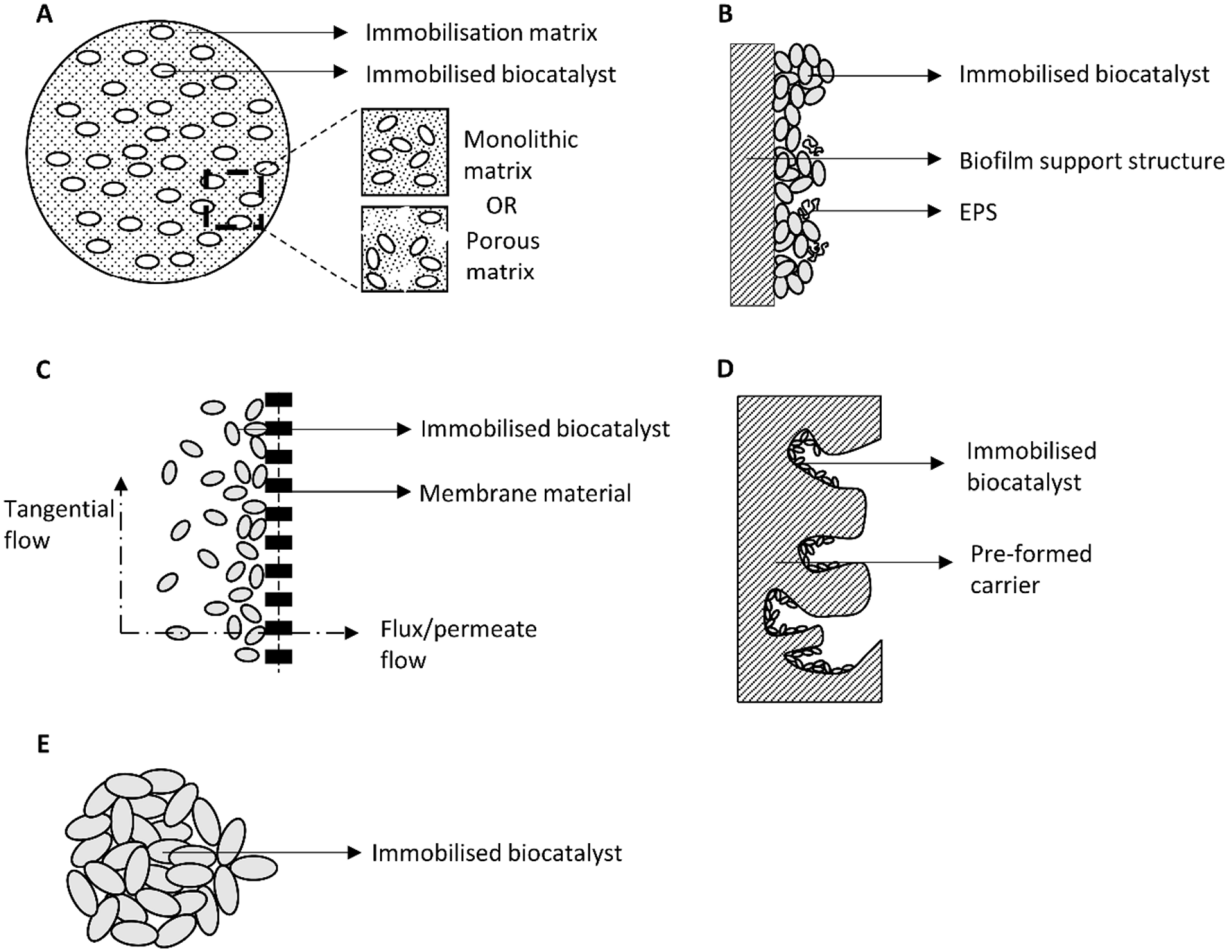
Schematic diagram of immobilisation via gel entrapment in either a monolithic matrix, or a porous matrix (**A**), via the formation of a biofilm (**B**), behind a membrane (**C**), within porous matrices (**D**) or via flocculation or aggregation (**E**)

The separation of the SRT and HRT within a PBR allows for efficient and controllable continuous operation and prevention of cell washout which is a major issue which operation using planktonic culture PBRs face. Biomass immobilisation refers to the physical confinement of a microorganism or a culture thereof into a localized area without loss of biological activity, partially separated from the bulk fluid medium but allowing for substrates to diffuse to the microorganism and products to diffuse outwards (Willaert 2018). Through immobilisation of biomass, the challenges associated with cell washout can be minimized or mitigated, as well as potentially improving upon other challenges faced with commercial applications of photosynthetic biocatalysts. Immobilisation of microorganisms can be achieved through entrapment within a solid material, adsorption onto preformed carriers, entrapment behind a barrier or self-aggregation of the microorganisms (Obradovic et al. 2004). These various forms of immobilisation aim to separate the biocatalyst from the liquid media and concentrate the biocatalyst to a localised volume, resulting in easy separation of the microorganisms from the bulk liquid media. A graphical summary of the types of immobilisation is provided by Fig. 1.

There are numerous advantages for the immobilisation of microorganisms (or parts thereof) over the use of planktonic cell cultures in PBRs. Biomass immobilisation may enhance the amount of light received by the biocatalyst, separate inhibitory or harmful products or by-products from the biocatalyst, control the biomass concentration in the bioprocess, protect cells against fluctuations in environmental conditions such as temperature, pH or shear stresses, and allow for ease of storage for future use (Dervakos and Webb 1991; Obradovic et al. 2004; Krasňan et al. 2016; Du Toit and Pott 2020). Although generally advantageous, careful consideration should be given to the type of photosynthetic microorganism and application since not all are suitable for immobilisation. Cellular immobilisation is generally not recommended for processes whereby primary metabolites are the desired products since they are directly associated with microbial growth which may be significantly affected by the physical confinement associated with certain forms of immobilisation (Bai et al. 2011). In addition, where biomass is the desired product, operating the PBR with an SRT < HRT may improve biomass productivity over operation where SRT > HRT, with the added advantage of reducing the water and nutrient consumption (Barbera et al. 2020). This type of operation may be suited towards biomass cultivation in membrane PBRs or by exploiting specific microorganisms’ abilities to self-aggregate and flocculate over immobilisation via biofilm formation or entrapment within solid matrices. Additional processing costs and complexities are also introduced through microbial immobilisation, which requires consideration towards the overall economic outcome of the bioprocess.

In addition to the type of microorganism, consideration should also be given to the immobilisation material, including the associated diffusion rates, strength, thermal and chemical stability, transparency, as well as the biocompatibility towards the immobilised microorganism. Although the rate limiting step in most photosynthetic bacteria is attributed to the dark reactions or light-independent reactions associated with the catabolism of organic matter (Hallenbeck and Benemann 2002), the design considerations for an immobilisation matrix should not introduce other possible rate limiting steps (such as diffusion of substrates to the organism). The immobilisation material should be stable in general bioprocessing conditions, including the proposed operating temperature, stresses involved in the bioreactor, as well as contact with the substrates or products involved which should not cause degradation of the immobilisation material. The material of choice should not require harsh gelation conditions or chemicals which could be detrimental to cell viability. The last major design consideration is specific towards the immobilisation of photosynthetic microorganisms where the material should be transparent and not limit the transfer of light to the entrapped cells.

Various PBR configurations have been developed which are possible for the use of immobilised biocatalysts, each with consideration towards the method of immobilisation. These different PBR designs and methods of immobilisation are discussed in the subsection to follow.

### Immobilisation within solid matrices

Biomass immobilisation or cell entrapment within solid matrices is a form of immobilisation whereby a culture of cells or cell components (such as enzymes) are entrapped within a material. Although immobilisation within a solid material generally takes the form of microbial culture suspended in the matrix held through physical confinement, covalent entrapment can also be used, generally employed for enzyme immobilisation, where chemical bonds form between the enzyme and polymeric immobilisation material (Tanwar et al. 2022). Immobilisation within solid materials is an effective method of immobilisation and has been employed using organic or inorganic carrier materials. This method of immobilisation can be further categorized into two separate categories based on the immobilisation material; namely: immobilisation through gel entrapment or onto pre-formed porous matrices (Baron and Willaert 2004).

Immobilised photosynthetic microorganisms within solid matrices have shown enhanced performance when employed in continuously stirred PBRs or fluidised bed PBRs over other configurations such as in a packed bed PBR or as a planktonic culture (Vincenzini et al. 1982b; Bettmann and Rehm 1984; Fißler et al. 1995; Lozinsky et al. 2003; Du Toit and Pott 2020; Ross and Pott 2021). The use of immobilised cells within solid matrices aims to tackle the challenges presented by planktonic cell systems, such as downstream separation requirements, mutual shading of microorganisms in biofilm or planktonic cell systems, and biomass washout (Wang et al. 2010). Due to the nature of the internal entrapment of this immobilisation method, special consideration should be given to the transparency of the immobilisation material employed, since an opaque material could significantly impede the biocatalyst performance and overall bioprocess efficiency. In addition to the material transparency, the material should also not impose mass transfer limitations, should be mechanically, thermally, and chemically stable as well as being biocompatible towards the immobilised microorganism.

#### Gel entrapment

Gel entrapment refers to the loading of biomass into a low porosity material for cell retainment while allowing for substrates to diffuse from the bulk solution into the material and to the cells, and for products and wastes to diffuse out of the immobilisation material (Obradovic et al. 2004). The gels can be formed into almost any form desired, although spherical beads are generally manufactured. Gel materials can be further classified into two classes, namely: natural polymers or synthetic polymers. Natural polymers include materials such as alginate, agar, carrageenan, cellulose, or chitosan while synthetic polymers include materials such as polyacrylamide, polyvinyl alcohol (PVA), latex, or polyethylene glycol, amongst various others (Berillo et al. 2021). These polymers or combinations thereof are suitable materials for immobilisation matrix use since they are generally biocompatible, simple to produce, mechanically stable, are low cost and are easily modifiable to manipulate various physical properties.

In application for photosynthetic microorganisms, the transparency of the resulting gel carrier is an important property to consider, amongst the other physical properties such as diffusivity, mechanical strength, biocompatibility, thermal stability, and chemical resistance. These physical properties can be modified through careful selection of the type and concentration of polymer and possible crosslinker, cosolvent or copolymer, as well as the gelation conditions and procedure.

The entrapment of biomass within gels is generally done through the addition of a cell culture to a liquid solution of the immobilisation material, followed by a gelation process induced through various mechanisms labelled as being either chemical or physical crosslinking. Chemical crosslinking refers to the covalent bonding of adjacent polymer chains through a variety of different chemical reactions including free-radicle polymerization, complementary group reactions, radiation and enzyme-catalysed reactions, forming irreversible hydrogels (Li et al. 2014; Kamoun et al. 2015; Bi and Liang 2016). Toxic chemicals or harsh gelation conditions are generally required for the formation of chemically crosslinked hydrogels. Crosslinking chemicals including glutaraldehyde, boric acid and tannic acid are toxic to microorganisms and as a result, are generally not suitable in biotechnical applications (Dong et al. 2018; Song et al. 2021). The alternative option to chemically crosslinked hydrogels is physically crosslinked hydrogels, formed through the entanglement of polymer chains or through hydrophobic interactions in copolymeric systems, ionic forces, crystallization or hydrogen bonding between polymer molecules (Hoffman 2002; Minhas et al. 2013).

Agar and alginate have been used extensively for cell immobilisation due to their desirable biocompatibility, transparency, and diffusivity characteristics. Apart from these desirable characteristics, they show rather low mechanical strength as well as alginate being prone to dissolution in the presence of monovalent cations, placing limitations on their use in bioprocessing conditions or using common wastewaters as feedstocks (Bucke and Brown 1983; Smidsrød and Skjåk-Bræk 1990; Garbayo et al. 2002).

Polyacrylamide gels show good mechanical strength and chemical stability but are often not biocompatible due to the denaturing effect that the monomers and free radicals produced during the polymerization reactions have on the microorganisms during the gelation procedure (Bucke and Brown 1983). Polyvinyl alcohol (PVA) cryogels have been shown to be an excellent immobilisation material for photosynthetic microorganisms due to their biocompatibility, minimal resistance to diffusivity, mechanical strength, chemical stability, ease of production and transparency at optimal PVA and co-solvent solution concentrations (Holloway et al. 2013; Du Toit and Pott 2020). Building upon previous work, a display of the extent of manipulation of PVA cryogels through the modification of the method of cryogelation and the composition of species and addition of a co-solvent or cryoprotectant within the cryogel is shown by Du Toit and Pott (Lozinsky and Plieva 1998; Du Toit and Pott 2020). A transparent and mechanically stable PVA cryogel was developed after a single freeze–thaw cycle through the addition of glycerol as a cosolvent in this study (Du Toit and Pott 2020), which can be compared to previous PVA cryogels which were opaque and required multiple freeze–thaw cycles to create a mechanically stable cryogel (Lozinsky et al. 1995; Lozinsky and Plieva 1998; Du Toit and Pott 2020).

An example of where gel-entrapment has been beneficial is the photofermentation of hydrogen by *R. palustris.* Due to the ability of *R. palustris* to produce hydrogen in the stationary phase, cell-entrapment of this purple non-sulfur bacteria in gels has shown great potential for biohydrogen production (Wang et al. 2010; Du Toit and Pott 2020). The immobilisation of this bacteria in transparent PVA-based cryogels and employed in a fluidised bed and continuously stirred PBR have shown to produce hydrogen at a specific production rate of approximately 11 mL_H2_ g^−1^_CDW_ h^−1^ (Du Toit and Pott 2020; Ross and Pott 2021). Although this may be relatively average in terms of production values obtained using a fed-batch PBR configuration, it is higher than that obtained when alginate is used as the immobilisation material which resulted in a specific production rate of 3.7 mL_H2_ g^−1^_CDW_ h^−1^ (Fißler et al. 1995; Tsygankov and Khusnutdinova 2015).

Apart from the immobilisation of *R. palustris* in PVA gels, various other photosynthetic microorganisms including *R. capsulata, C. thiosulfatophilum* and *R. rubrum* have been immobilised in other materials including ionic crosslinked carrageenan hydrogels, ionic crosslinked calcium alginate hydrogels and agar hydrogels. Immobilisation of these bacteria have been employed in these cases for predominantly the photofermentative production of hydrogen and bioremediation of organic wastes, but as well as the removal of hydrogen sulfide from a gas stream in the case of *C. thiosulfatophilum* (Vincenzini et al. 1982b; Francou and Vignais 1984; Fißler et al. 1995; Zhang et al. 2017).

In addition to entrapment within gels, nano-porous latex coatings can be used as an immobilisation material as well for the entrapment of photosynthetic microorganisms. Immobilisation in latex coatings addresses the challenges faced with other immobilisation materials or methods. These challenges include the lack of an adhesive immobilisation coating, high biomass loadings, inexpensive immobilisation material, biocompatible material and immobilisation procedure and a mechanically stable immobilisation matrix (Lyngberg et al. 2001; Gosse et al. 2007). The thickness of latex coatings containing immobilised cells can be tightly controlled at very thin ranges (< 100 µm) which is advantageous when optimising the balance between uniform light transmittance, diffusivity, and biomass concentration (Gosse et al. 2007).

*R. palustris* was immobilised in a thin layer nano porous latex coating by Gosse et al. (2007), exhibiting good biocompatibility and highlighting the effect in which the latex coating thickness had on the hydrogen production. Under optimal conditions, this PBR configuration resulted in the specific biological hydrogen production equal to 0.55 mol of hydrogen in a time of 120 h or an average of 1.92 mL_H2_ mL_Material_^−1^ h^−1^. An advantage towards the employment of latex coatings for the immobilisation of microorganisms is seen by the ability to easily produce mechanically stable nanoporous coatings with tight controllability around the film thickness (Flickinger et al. 2007; Gosse et al. 2007).

The gelation process of latex films requires latex polymeric materials such as styrene or butyl or styrene acrylics with different additives including texanol changing the process and manipulating the produced film (In-na et al. 2022). In addition to the chemical formulation of the latex film, the gelation conditions also affect the outcome of the film, with higher gelation temperatures producing a harder but less adhesive film while films formed below the glass temperature generate softer films but with stronger adhesive properties (In-na et al. 2022). In the study by In-na et al. (2022), cyanobacteria were immobilised in latex films applied to loofah sponges. The study showed carbon dioxide capture over several months without significant loss of biocatalyst from the material.

When thin layer latex films are used as an immobilisation material, the diffusion of substrates into the material should be considered since latex material is inherently relatively resistant to diffusion of chemical species. Relative diffusion rates are expressed as the diffusion of a chemical species through the specific material compared to through water. The relative diffusivity for latex are generally in the range of 0.0003 to 0.2 which is significantly lower than that generally obtained with PVA cryogels which are often in the range of 0.7 to 0.9 for compounds such as glycerol and glutamate (Lyngberg et al. 2001; Du Toit and Pott 2020). Alginate exhibits a relative diffusivity of common carbon sources of approximately 0.85 (Øyaas et al. 1995; Garbayo et al. 2002). The diffusion of substrates and products to and from the immobilised biocatalyst can be manipulated through optimization of the latex layer thickness, which is readily controlled though the formation procedure. In addition to possible diffusion limitations, the application of latex as an immobilisation material should take into consideration the chemical constituents of the feedstock. Latex may degrade or dissolve in the presence of solvents and as such, its application in challenging waste streams may be limited.

A summary of the various types of materials commonly used and employed for the physical entrapment of photosynthetic microorganisms amongst others is provided in Table 1. This table refers to relevant information and mechanical properties including the transparency, the mechanical strength of the material, the chemical and thermal stability, the diffusivity as well as the biocompatibility of the material. These factors and physical properties have implications on the suitability for use of the material with respect to certain photosynthetic microorganisms in specific bioprocesses or biotechnical application.

**Table 1:** Summary table of common types of materials used for entrapment of microorganisms

Material	Gel type	Crosslinking chemical	Micro-organism	Photo-trophism	Transparency	Mechanical strength	Chemical stability	Thermal stability	Bio-compatibility	Diffusivity	Reference
PVA	Chemically crosslinked hydrogel	Boric acid	*R. palustris, R. sphaeroides* NR-3	Yes	Good	Good	Good		Fair	Good	Nagadomi et al. (2000)
PVA	Cryogel	None	*Z. mobilis*	No	Poor	Excellent	Excellent			Good	Lozinsky et al. (1995)
PVA	Cryogel	Glycerol	*R. palustris* NCIB 11774	Yes	Excellent	Excellent	Excellent	Very good	Excellent	Good	Du Toit and Pott (2020), Ross and Pott (2021)
PVA	Hydrogel	LentiKat solution	*S. cerevisiae*	No		Good		Good	Excellent	Good	Krasňan et al. (2016)
Alginate	Ionic crosslinked hydrogel	Calcium or strontium	*S. cerevisiae, fibroblasts, R. palustris DSM 131, R. rubrum, R. capsulata, C. thiosulfatophilum* ATCC 17092	Both	Good	Fair	Poor		Very good	Very good	Smidsrød and Skjåk-Bræk (1990), Woo Kim and Nam Chang (1991), Fißler et al. (1995), Garbayo et al. (2002), Rakin et al. (2009), Kaklamani et al. (2014), Zhang et al. 2017)
Agar	Hydrogel	None	*R. rubrum G-8 BM & 7061, R. capsulata, R. palustris 42 OL, Protease enzyme*	Both	Good	Poor	Fair	Good	Good	Fair	Vincenzini et al. (1982b), Hirayama et al. (1986), Planchard et al. (1989), Banerjee and Bhattacharya (2011), Samprovalaki et al. (2012), Zhang et al. (2017), Sattar et al. (2018)
Chitosan	Chemically crosslinked hydrogel	Sodium dodecyl sulfate, glutaraldehyde, sulfuric acid or formaldehyde	None	No	Fair	Fair	Good	Fair	Good	Fair	Yang et al. (2004), Kaushal et al. (2018), Dwamena et al. (2020)
Chitosan	Hydrogel	None	None	No	Fair	Fair	Poor	Fair	Good	Good	Zhu et al. (1999), Nie et al. (2016), Eremina et al. (2019), Chamorro Rengifo et al. (2019)
Carrageenan	Ionic crosslinked hydrogel	KCl	*C. vulgaris, R. palustris* DSM 131*, R. rubrum* G-9 BM*, R. capsulata* B10	Yes	Good	Poor	Poor	Good	Good	Good	Francou and Vignais (1984), Hirayama et al. (1986), Fißler et al. (1995), Lau et al. (1998), Popescu and Boscornea (2007)
Latex	Thin layer	None	*R, palustris* CGA 009*, E. Coli* HB 101	Both	Good	Good	Excellent	Good	Excellent	Poor	Lyngberg et al. (2001), Song et al. (2001), Gosse et al. (2007)

#### Pre-formed carriers

Immobilisation of microorganisms on porous pre-formed materials occurs predominantly through the attachment of cells to the surface structure of the support material and through collection in blind pores directly from the inoculated medium (Baron and Willaert 2004). This method of immobilisation is advantageous since it does not require the addition of cross-linking or entrapment chemicals, the support material is chemically and metabolically inert, sterilization and reuse are relatively easy, and a high percentage of cell viability is retained through this immobilisation method. Although this method of immobilisation is advantageous in many regards, it does come at the cost of a decrease in loaded cell concentration compared to gel entrapment (Yokoi et al. 1997; Baron and Willaert 2004). This is since the bioactive area is limited to internal pores rather than the entire volume of carrier material as with immobilisation via gel entrapment. This method of immobilisation is also limited to photosynthetic microorganisms which can form biofilms or clusters within internal pores of the material, to maintain the immobilised biomass concentration.

Organic and inorganic materials have been employed as pre-formed biomass carrier matrices, each with their respective advantages and disadvantages or suitability toward a specific photosynthetic microorganism or bioprocessing aim. These materials include materials such as polyurethane, polyvinyl foam, plastics, cellulose and other polymers as well as inorganic materials such as glass, silica and ceramics (Mavituna 2004). Many of these materials are non-transparent, which may be a significant limitation in photosynthetic systems.

The biomass containing materials can be used in PBRs configured in a similar fashion as would be used for gel entrapped biomass i.e., in a packed bed or fluidised bed/continuously stirred PBR. Yokoi et al. (1997) investigated the loading of heterotrophic bacteria *Enterobacter aerogenes* onto various support structures including chitosan beads, cellulose-foam carriers and porous glass beads through adhesion onto the material. Although *E. aerogenes* is not a phototrophic bacterium, the results showed that immobilisation on porous glass beads resulted in the highest hydrogen production rate due to the high diffusivity associated with porous glass beads and its high specific gravity, promoting the beads to stay in suspension rather than floating to the surface of the medium at higher flow rates. The use of glass beads as an immobilisation material does come with the pitfall of the cost involved with manufacturing or purchasing glass and the abrasion effects that the beads have on one another within the bioreactor, accelerating the degradation process of the physical beads and increasing the operating cost for the bioprocess.

Fedorov et al. (1998) investigated the employment of porous polyurethane foam as an immobilisation carrier for the purple non-sulfur bacteria *Rhodobacter sphaeroides* in a continuous flow PBR. Using lactate as a carbon source and under optimized conditions, the hydrogen production conversion efficiency was equal to 86% with production rate of 0.21 mL_H2_ mL_Material_^−1^ h^−1^. The conversion efficiency obtained in this study was higher than that obtained when *Rb. sphaeroides* was immobilised on porous glass sheets (Tsygankov et al. 1994). In that study a conversion efficiency of 75% was reached with a hydrogen production rate of 1.3 mL_H2_ mL_Material_^−1^ h^−1^ porous glass at the same lighting intensity, although a higher specific dry cell loading of 11.2 mg mL^−1^ of porous glass was used (Tsygankov et al. 1994). An advantage of immobilisation on polyurethane foam over porous glass beads or other similar materials is their low associated cost and the additional complexities associated with porous glass including fluidisation velocities and its brittleness.

In another example, photosynthetic bacteria including two strains of *Rhodobacter sphaeroides* and *Rhodopseudomonas palustris* were immobilised onto porous ceramic beads for the bioremediation of a synthetic waste stream containing organic contaminants, phosphate, nitrate and hydrogen sulfide in an air lift PBR (Nagadomi et al. 2000). The PBR was operated under aerobic, illuminated conditions and showed efficient removal of these contaminants when operated in batch and semi-continuous treatment conditions. During batch treatment, the removal efficiencies of COD, phosphate and nitrate were 42.1%, 75.0% and 98.7%, respectively. These efficiencies were improved in semi-continuous treatment where the removal efficiencies of COD, phosphate and nitrate were equal to 98.8%, 95.0% and 99.9%, respectively (Nagadomi et al. 2000).

Immobilisation of photosynthetic microorganisms on pre-formed porous matrices tends to show similar production rates to cell entrapment within gels when looking at the use of photosynthetic bacteria for hydrogen production. These similar values in hydrogen production across the different methods of immobilisation within solid matrices are likely due to similarities in PBR configuration and the comparative advantages and disadvantages which gel entrapment and immobilisation within porous matrices have to each other. While gel entrapment may have a transparency or light transmittance advantage over entrapment within a porous material, its resistance to mass transfer may come at a disadvantage. A higher biomass loading can be obtained in gel entrapment compared to immobilisation on a porous matrix while the effect that the immobilisation procedure during entrapment in a gel might adversely affect the cells compared to the inert method of immobilisation for entrapment on a porous material.

The algal species, *Prototheca zopfii* was immobilised in pre-formed polyurethane foam carriers as a method of immobilisation for the degradation of alkanes in a bubble column reactor (Yamaguchi et al. 1999). Immobilised cells or *P. zopfii* exhibited a two-fold higher degradation rate of n-alkanes compared to cells entrapped within calcium alginate hydrogels. The results of this study are supported by Travieso et al. (1996) where the green algae, *Chlorella vulgaris, Chlorella kessleri* and *Scenedesmus quadricauda* were immobilised in pre-formed polyurethane foam carriers. These green algae were employed for the bioremediation of a waste stream and nutrient removal. Although the results obtained from the microbial consortium immobilised in alginate hydrogels were promising, similar results were obtained from the green algae immobilised in the pre-formed polyurethane support structure (Travieso et al. 1996).

### Biofilm formation

Biofilm PBRs have been employed in a variety of applications including wastewater treatment (Muffler et al. 2014; Gao et al. 2015, 2016; Katam et al. 2021), lipid production (Economou et al. 2015), hydrogen production (Tian et al. 2010) or algal cultivation (Ozkan et al. 2012). In a similar application to immobilisation onto pre-formed porous surfaces, immobilisation through biofilm formation is considered as the natural attachment of microorganisms to a set area or surface in a PBR. From the literature it could be identified that the photosynthetic microorganisms that have been employed in biofilm PBRs thus far include purple non-sulfur bacteria, cyanobacteria and algae.

The formation of biofilms occurs in three distinct phases namely, cell attachment, colonization with extracellular polymer substance (EPS) production followed by maturation of the biofilm (Tsygankov and Kosourov 2014). Cell attachment refers to the adhesion of microorganisms to the surface of the material, with the attachment dependent on the material surface properties, fluid flow properties and the deposition rate of the microorganism onto the surface (Tsygankov and Kosourov 2014). The second phase of biofilm formation refers to the growing and reproduction of cells which are irreversibly attached to the surface during the cell attachment phase. The reproduction of attached microorganisms results in the formation of microcolonies on the surface with cells continuing to synthesize EPS, predominantly extracellular polysaccharides. These extracellular polysaccharides play a major role in the formation of mature biofilms through the promotion of cell adherence to surfaces and cells, protection from environmental stresses and structure enhancement, acting as a biomolecular glue and communication channel between cells as well as to the intended immobilisation surface (Bazaka et al. 2011; Ferreira et al. 2011; Limoli et al. 2015). Indeed, the biology of biofilm formation is rather complex, and different species behave in various ways—some will readily form biofilms, while some require a ‘trigger’ (such as quorum sensing in *R. palustris*) (Schaefer et al. 2008; Ehrlich et al. 2023). During the colonization phase, the microorganism concentration increases and intercellular communication becomes possible with a shift in cellular mass behaviour with respect to biofilm structure and metabolic activity at a critical concentration dependent on the microorganism population density (Tsygankov and Kosourov 2014). The final stage of biofilm formation is characterized by the maturation and increase in cell density of the biofilm (Tsygankov and Kosourov 2014; Ehrlich et al. 2023). During this stage, the continuous growth of the biofilm forms a complex three-dimensional structure with the thickness dependent on a few factors including the microorganism, age, substrate availability and hydrodynamic or fluid flow properties. As the biofilm ages and thickness increases, the rate of cell detachment and concentration of planktonic cells in the medium increases to reach a steady-state point with the rate of biofilm formation. At this point, the biofilm is in an equilibrium and significant changes in the thickness or size of the biofilm is not expected with constant environment factors.

The natural immobilisation of microorganisms through the formation of biofilms may be advantageous in the long term over other forms of immobilisation. This is because it is a natural process without the requirement for toxic gelation materials where the cells and their three-dimensional cellular complex can adapt to the environmental conditions through the formation and adaptation of a biofilm (Muffler et al. 2014). In contrast to this advantage, immobilisation through biofilm formation generally takes a considerable amount of time to form a stable biofilm. The first phase of biofilm formation is generally the most time-consuming stage, although it can be accelerated through surface modification or suitable surface support material selection (Tsygankov and Kosourov 2014).

This immobilisation technique requires a photosynthetic microorganism with the ability to form a biofilm and a dedicated area within the PBR for adhesion or attachment of cells to occur. To increase adhesion area which in turn increases the area of biofilm created, multiple different PBR geometries and inserts have been developed. These applications to increase cell attachment area include the addition of packing in various forms to the reactor volume including plastic structures, transparent sponge, glass beads, silicone, glass rods suspended in the reactor or through the addition of grooves to the reactor walls (Qureshi et al. 2005; Tian et al. 2010; Zhang et al. 2010; Economou et al. 2015; Strieth et al. 2021; Katam et al. 2021). The introduction of additional surface area within the PBR allows for larger biofilm development and biomass growth, increasing productivities or yields. The light penetration to within the PBR should be considered since the effects of mutual shading increases with increasing biomass concentration with the additional packing.

The investigation conducted by Tian et al. (2010) investigated the use of a biofilm PBR for biohydrogen production using the purple non-sulfur bacterium *Rhodopseudomonas palustris*. In this PBR the biofilm was created by inducing the bacteria to attach to glass beads that were packed inside the PBR. The goal of this PBR was to utilize synthetic wastewater to produce biohydrogen under the illumination of LEDs at an intensity of 5000 lx. This PBR configuration resulted in a good hydrogen production rate of 38.9 mL_H2_ L^−1^ h^−1^. Although this PBR design resulted in a good hydrogen production, it showcased the typical disadvantage associated with biofilm PBRs as the duration of startup prior to steady-state hydrogen production and biofilm formation was 60 days.

An investigation by Wang et al. (2019) showed that by increasing the surface area in the PBR, a higher hydrogen production rate could be attained through the use of a grid columnar flat panel PBR. The high hydrogen production rates achieved are due to higher specific biomass concentrations resulting from larger surface areas available for biomass attachment through the introduction of a grid structure and enhanced mass transfer between the medium and the biofilm. This indicated not only the importance of a larger surface area but also the interaction of biofilm and the liquid medium or efficient mixing inside the PBR. This result was reiterated by Zhang et al. (2010) where a groove-type PBR performed better than a flat panel due to increased surface area and enhanced mass transfer.

In another application by Economou et al. (2015), the filamentous cyanobacterium *Limnothrix* sp. Was used for the simultaneous treatment of wastewater and lipid production in a biofilm PBR. In the PBR, glass rods were available to increase the surface area for cyanobacteria attachment and growth. It was observed that for filamentous cyanobacteria, the specific biomass productivity per surface area was equal to 1.11 g m^−2^ day^−1^ or a total biomass productivity of 38.77 mg L^−1^ day^−1^ and intra-cellular lipid productivity equal of 8.14 mg L^−1^ day^−1^ which was almost double that of a free cell system, due to an increase in attachment surface area. This result was reiterated by Johnson and Wen (2010) for a *Chlorella sp.* algal biofilm PBR, however in this investigation the aim was to test the effect of the attachment of biofilm algae to different materials. This study showed that the algal cultures of *Chlorella* favoured attachment to polystyrene structures over the other structures tested, including cardboard, polyethylene landscape fabric, loofah sponge, polyurethane foam, and nylon sponge (Johnson and Wen 2010).

The exploitation of the ability of specific microorganisms to naturally form biofilms can be extrapolated to the application of terrestrial microorganisms in PBRs for their cultivation (Strieth et al. 2021). The study done by Strieth et al. (2021) highlighted the fact that biofilms and their formation are complex by nature, whether the medium be aerosol- or liquid-based. The secretion of various EPSs interacts with different packing materials differently and the excretion of various cellular biproducts may affect the surface chemistry of the cells, packing material and their interaction. In addition to the type of microorganisms, consideration towards the materials hydrophobicity, contact angle, light as well as the temperature distribution and transfer coefficients is required. This study observed that variation in similar packing materials did not significantly affect the cultivated biomass concentration, but the type of material did have an effect on the thickness of the biofilm.

In summary, a major drawback to using biofilm PBRs is a long required initial HRT and start-up period, in comparison to planktonic cell systems. This extended start-up period is a requirement for the attachment of cells to the material surfaces. The attachment of cells to structure walls also decreases their exposure to substrates and nutrients in the medium, although this disadvantage can be minimized through advanced reactor design including packing structures and efficient mixing (Economou et al. 2015; Wang et al. 2019). In conjunction to the biofilm attachment, the exposure to light is significantly decreased by the attachment of microorganisms to the PBR walls resulting in mutual shading. Depending on the quality of effluent required from the PBR, the rate of detachment of microorganisms from the surface material should be taken into consideration as the concentration of cells in the effluent may require downstream removal. However, if cell detachment is negligible or not a concern, an advantage to this PBR type is the elimination of the downstream requirement for separation of the photosynthetic microorganisms and the liquid medium. This means that the majority of the biomass is retained for increased biomass growth rates and possibly achieving better conversion efficiencies through continuous operation where mature colonies have formed stable biofilm structures. Another benefit is that the microorganisms can be readily harvested from the packing or PBR surface for use in other products without the requirement to isolate from within an internal structure such as associated with immobilisation via entrapment within solid matrices which would be challenging.

### Membrane photobioreactors

The third method of HRT and SRT separation is through the containment of cells behind a barrier, which can be achieved through the use of a membrane (Obradovic et al. 2004). PBRs which integrate a membrane have been applied in waste stream bioremediation, or production of a valuable product such as lipid or biogas production, often using microalgae as the photosynthetic microorganism (Honda et al. 2012; Discart et al. 2014; Chang et al. 2018; Oosthuizen et al. 2021).

The use of membrane PBRs is advantageous in the case where molecules with large molecular weights present in the feed stream or specific products are unwanted and should be separated from the biocatalyst (El-Mansi et al. 2011). This can be a gentle immobilisation procedure since no toxic chemicals are required. The diffusion of certain compounds and the rate of mass transfer through the membrane can readily be manipulated by altering the fluid pressure or pore size in the membrane PBR. A disadvantage which is inevitable with membrane PBRs is the clogging effect which cells will have on the membrane pores and the detrimental effects that the high pressures and shear stresses have on the cell viability (Zou et al. 2022).

The synthetic polymeric material polymethyl methacrylate (acrylic plastic) is often used to construct the membranes for membrane PBRs although other materials such as chlorinated polyethylene, polyvinyl chloride and silica have also been used as a construction material. Generally, a hollow fiber or a flat-sheet membrane configuration are used in biotechnical applications. Hollow fiber membranes have been extensively employed, often in the wastewater treatment sector (Naim et al. 2021). They provide larger surface-to-volume ratios over flat-sheet membranes without the need for membrane support while flat-sheet membranes generally have simpler geometries, providing easier accessibility for configuration modification and maintenance (El-Mansi et al. 2011). Careful selection of the type of membrane and operating configuration is needed since the pore size is largely dictated by the microorganism employed. Smaller microorganisms would require a membrane material with smaller pores, introducing larger pressure differentials and resulting in shear stresses which could be detrimental to cell viability.

Numerous membrane PBRs have been developed for the cultivation and application of the green algae, *Chlorella vulgaris*. These applications include wastewater treatment (Marbelia et al. 2014; Gao et al. 2016), removal of toxic contaminants (Gao et al. 2016), production of biomass (Marbelia et al. 2014; Bilad et al. 2014; Gao et al. 2016), landfill leachate remediation (Chang et al. 2016, 2018), bio-lipid production (Chang et al. 2018) and carbon dioxide sequestration (Honda et al. 2012). These studies showed that the employment of green algae such as *Chlorella vulgaris* immobilised behind membrane PBRs tended to increase biological productivities, resulting in higher processing efficiencies for biomass cultivation or remediation of challenging waste streams compared to planktonic or batch PBRs.

The application of algae and algal consortia in membrane PBRs for the combined production of biomass and wastewater remediation exhibits itself as a potential solution to both the challenge of efficient wastewater treatment as well as the bioproduction of valuable products (Marbelia et al. 2014; Gao et al. 2016; Solmaz and Işık 2020). Optimised PBR operation allows for efficient nutrient removal from toxic effluents combined with the cultivation of microorganisms which can be readily harvested from an algal rich concentrate stream. These algae show promise as a potential future source of protein for humans or animals, biofuel, enzymes, pharmaceuticals or other valuable commodity chemicals (Feng et al. 2011; Varfolomeev and Wasserman 2011; Mobin and Alam 2017; Chew et al. 2017; Solmaz and Işık 2020).

Apart from the production of valuable biomass as health supplements or for wastewater treatment purposes, membrane PBRs have also shown promise in the production of biogas, and hydrogen in particular. The purple non-sulfur bacteria, *Rhodopseudomonas faecalis* was employed in a membrane PBR to produce hydrogen gas using acetate as the main source of carbon (Xie et al. 2012). The bacterial biomass in its stationary phase was retained in the PBR using a dead-end semi-permeable, cellulose acetate membrane with a pore size of 0.22 µm. A maximum specific hydrogen production rate of 38.61 mL h^−1^ g_CDW_^−1^ was observed at continuous operation. This hydrogen production rate is relatively high compared to those obtained through immobilisation in solid materials which are generally in the range of 8–15 mL_H2_ g_CDW_^−1^ h^−1^, although values of up to 54 mL_H2_ g_CDW_^−1^ h^−1^ have been observed when *R. capsulata* was immobilised in carrageenan beads (Francou and Vignais 1984; Du Toit and Pott 2020; Ross and Pott 2021).

The use of membrane PBRs for the treatment of domestic wastewater or pre-treated sewerage through the use of a consortium of purple photosynthetic bacteria has been shown to be a promising and efficient approach (Hülsen et al. 2016; González et al. 2017). These bacteria make use of nutrients available in the wastewater including nitrogen-, phosphorous- and sulfur-containing compounds and dissolved organic matter, effectively removing them from the effluent and increasing the quality of the final effluent stream, possibly for reuse or discharge. In order to be used in this way, the solids (the biomass) must remain in the PBR while the wastewater travels through the PBR as it is treated—an excellent example of separating SRT from HRT to good effect. The bacterial community identified on one of these membranes included species of the *Rhodopseudomonas* genus including *faecalis* and *pseudomonas* as well as *Chyseoabacterium rhizoplanae* when fed with the effluent from an anaerobic membrane bioreactor (González et al. 2017). When the feed source was domestic wastewater, a variety of photosynthetic bacteria as well as non-photosynthetic bacteria was identified (Hülsen et al. 2016). The photosynthetic bacteria identified included species from the *Rhodobacter, Rhodocyclus, Rhodopseudomonas, Allochromatium* and *Thiocystis* genera. These two separate studies both showed that the use of an anaerobic membrane PBR can efficiently treat a variety of wastewater to discharge standards in a single step. They demonstrate the use of photosynthetic bacteria and membrane PBR for industrial wastewater treatment and nutrient recovery applications. These studies also demonstrated that long term operation is feasible due to the relative ease of cleaning the membranes and their reusability, which has been improved through improved materials.

### Flocculation or aggregation

Self-aggregation or flocculation of cells can be naturally or artificially induced through the natural tendency of certain cells to clump together or through the addition of a chemical flocculant (Obradovic et al. 2004; Lu et al. 2019). The tendency of cells to naturally self-aggregate, also called auto-flocculation, is largely affected by the pH of the solution, sodium concentration, light intensity, inoculum size and the temperature (Lu et al. 2019), it can also be induced in some organisms through quorum sensing (Schaefer et al. 2008).

Lu et al. (2019) showed that the optimal conditions for the auto-flocculation of the photosynthetic bacterium, *Rhodobacter sphaeroides* was at a bioreactor temperature of 30 °C, a pH of 9.5, sodium concentration of 0.067 mol L^−1^, inoculum size of 420 mg L^−1^ and a light intensity of 5000 lx. These optimal conditions resulted in a flocculation ratio of 85% which is expressed as the percentage of biomass which forms flocs compared to what was originally a planktonic culture in the medium. At a biological scale, the cause for cells to self-aggregate is through the production of complex extracellular polymeric substances (Sheng et al. 2010; Lu et al. 2019). Apart from their role in the biofilm formation process previously mentioned, they play a major role in the formation of stable cell aggregates. This occurs through modification of cell and aggregate properties such as the surface properties and ability of cells to bind to each other, mass transfer properties, stability of the three-dimensional complex amongst other properties (Sheng et al. 2010).

Apart from natural flocculation, the addition of chemical flocculants can greatly increase the flocculation rate and efficiency, in a process termed artificial flocculation. Various flocculation agents have been employed for the immobilisation or flocculation of biomass including inorganic, organic and microbial flocculants. Organic flocculants are often advantageous as they are fast acting and often do not require large dosages. Some synthetic organic polymer flocculants pose serious negative health and detrimental environmental effects (Luvuyo et al. 2013). Some organic flocculants include materials such as chitosan, polyacrylamide, polyethylene amine (Moreno-Garrido 2008; Luvuyo et al. 2013; Li et al. 2020). Inorganic flocculants generally include salts of aluminium such as poly-aluminium chloride and aluminium sulphate as well as salts of iron including ferric chloride and ferric sulphate, amongst others (Luvuyo et al. 2013; Chatsungnoen and Chisti 2019). Although salts of aluminium, iron and zinc can provide efficient flocculation of microalgae as summarized by Chatsungneon and Christi (2019), these salts would often end up in the natural environment, causing detrimental environmental effects via soil and water pollution (Li et al. 2020), or in the biomass, which may impact or limit applications.

An attractive option for active flocculation is to use chitosan as the flocculation agent, as it is biodegradable, evading the challenge of flocculants being non-biodegradable and having negative effects on the environment and the organisms in the floc. Chitosan addition has proven to be an efficient method for flocculation of various microalgae from a solution of suspended cells (Maćczak et al. 2022), although it is still a relatively expensive material to exploit in processing (Acosta-Ferreira et al. 2020). Flocculation efficiencies of above 75% were obtained by Lubian (1989) when chitosan was used at concentrations in the range of 40–80 mg L^−1^ while Gualtieri et al. (1985) achieved flocculation efficiencies of up to 98% when a chitosan concentration of 200 mg L^−1^ was used. The results from this study were supported by the study done by Acosta-Ferreire et al. (2020) where chitosan was used to flocculate a microbial consortium including *Scenedesmus sp., Chlorella sp., Schroderia sp., and Chlamydomonas sp.* The microalgae removal using chitosan as a flocculating agent resulted in 99.2% removal efficiency (Gualtieri et al. 1985; Lubián 1989; Acosta-Ferreira et al. 2020).

A disadvantage associated with the use of natural or chemical flocculation for immobilisation is the relative weak strength of the microbial clusters and their fragmentation when exposed to shear stresses (Klausen et al. 2004). Shear stresses are imposed through hydrodynamic forces in PBRs by impeller mixing in the bulk fluid phase or fluid motions associated with the gas–liquid interface if gas-sparging is used (Hua et al. 1993). These shear stresses should be minimized to prevent fragmentation of the microbial flocs through weaker stirring or lower gas flow rates, possibly at the expense of decreased mass transfer rates and overall PBR performance. The aggregation of cells is also not preferred for immobilisation of photosynthetic microorganisms since they induce significant mutual shading between flocs and to cells within aggregates.

Although flocculation or self-aggregation of cells is a method of immobilisation, it is generally used as an improvement towards efficient separation of the solid biocatalyst from the bulk liquid medium. It is also often combined with a gravity-based separation procedure such as settling or centrifugation to enhance or accelerate the separation process. Flocculation is generally not used as an immobilisation method for photosynthetic organisms due to the mutual shading effect which clusters have on each other and the high sensitivity to shear which these clusters experience. As a result, very little information regarding immobilisation via self-aggregation or flocculation to enhance continuous bioprocess operation is available.

### Summary and applications

Although limited industrial applications currently exist in the large-scale application of photosynthetic microorganisms, notable research has been completed with regards to the separation of HRT and SRT in PBRs. This review investigated separation techniques including immobilisation via entrapment within solid matrices, through the formation of biofilms, behind a membrane and flocculation or aggregation. A common biotechnical application for photosynthetic microorganisms is for the production of biofuels, biogas or hydrogen using photosynthetic microorganisms commonly cultivated including green algae, cyanobacteria and purple non-sulfur bacteria.

A variety of purple non-sulfur bacteria and green algae have highlighted themselves as biological chassis organisms for exploitation in various applications. These applications include the bioremediation of challenging waste streams, sequestration of carbon dioxide, production of biomass, or to produce high purity hydrogen. Hydrogen is a high value commodity chemical and possible green energy alternative to fossil fuel-based energy. Purple non-sulfur bacteria including species of *Rhodobacter, Rhodospirillum* and *Rhodopseudomonas* have garnered much attention for their metabolic ability to produce hydrogen under specific conditions. These species have responded positively to various immobilisation methods to produce hydrogen and comparisons can be drawn between the hydrogen production of these species immobilised using different methods as explored in Table 2. Although comparisons are made in this table between the hydrogen producing capabilities of purple non-sulfur bacteria, there exist many different applications of different photosynthetic microorganisms for other applications including bioremediation of waste, biomass production or to produce valuable materials. These various different photosynthetic microorganisms employed for various applications may not provide a direct comparison from which insight can be drawn and will thus not be directly compared with each other.

**Table 2:** Hydrogen production potential of photosynthetic bacteria immobilised within solid materials

Immobilisation technique	Immobilization material	Microorganism	Hydrogen production (mL_H2_.g_CDW_^−1^.h^−1^)	Hydrogen production (mL_H2_.mL_Material_^−1^.h^−1^)	Source
None	None	*R. palustris* NCIB 11774	6.25	–	Du Toit and Pott (2020)
Gel entrapment	PVA cryogel (fluidised bed)	*R. palustris* NCIB 11774	8	0.112^a^	Du Toit and Pott (2020)
Gel entrapment	PVA cryogel (fluidised bed)	*R. palustris* NCIB 11774	15.74	0.22^a^	Ross and Pott (2021)
Gel entrapment	PVA cryogel (packed bed)				Ross and Pott (2021
Gel entrapment	Latex	*R. palustris* CGA 009	–	1.92^a^	Gosse et al. (2007)
Gel entrapment	Carrageenan	*R. capsulata* B10	54	0.313^a^	Francou and Vignais (1984)
Gel entrapment	Carrageenan	*R. rubrum* G-9 BM	18.04^a^	0.036	Hirayama et al. (1986)
Gel entrapment	Agar	*R. palustris* 42 OL	42	0.016^a^	Vincenzini et al. (1982b)
Gel entrapment	Agar	*R. rubrum* 7061	217	0.217^a^	Planchard et al. (1989)
Gel entrapment	Alginate	*R. palustris* DSM 131	0.067^a^	–	Fißler et al. (1995)
Pre-formed carrier	Polyurethane foam	*R. sphaeroides* GL-1	130	0.21^a^	Fedorov et al. (1998)
Pre-formed carrier	Porous glass	*R. sphaeroides* RV	116	1.3^a^	Tsygankov et al. (1994)

^a^Calculated from the original values provided from the source

Apart from immobilisation in solid matrices, immobilisation through the use of biofilms and membranes in PBRs have also shown potential in hydrogen production using photosynthetic bacteria. *R. palustris* immobilised in a biofilm PBR exhibited a hydrogen production of 38.9 mL L_Reactor_^−1^ h^−1^ (Tian et al. 2010) while *R. faecalis* immobilised using a membrane PBR exhibited a hydrogen production rate of 32.82 mL L_Reactor_^−1^ h^−1^ (Xie et al. 2012). A major limiting factor for the cultivation and harvesting of biomass is their separation or dewatering from the medium (Winck et al. 2016). Methods including filtration, centrifugation, sedimentation, flocculation or flotation or combinations of these methods have been previously employed to concentrate the biomass to a biomass rich stream (Dassey and Theegala 2013; Wang et al. 2014; Santo et al. 2023).

## Conclusions

A major challenge faced with PBRs is the risk of cell washout at higher dilution rates (particularly with relatively slow growing photosynthetic organisms), forcing the engineer to operate these bioreactors in batch or fed-batch operation mode. Recent technological advances have allowed for the separation of the hydraulic retention time and the solid retention time. These advances have driven progress towards continuous operation at a larger scale, improving the economic and technical feasibility of large scale or industrial implementation. A promising method of separation is to use immobilisation techniques where microorganisms are physically confined to a localized area (within the PBR) without the loss of biological activity. Advantages of immobilisation of photosynthetic microorganisms include a more uniform and efficient light distribution, higher controlled biomass concentrations, an extent of protection from environmental conditions and easy separation of the biocatalyst from the bulk fluid.

Various forms of immobilisation exist, namely entrapment within solid matrices, entrapment behind a membrane, self-aggregation or adsorption onto a pre-formed carrier. The mechanics, advantages, disadvantages and examples of these different immobilisation techniques were investigated with reference to that reported in the literature, bioprocess performance and efficiencies using photosynthetic microorganisms as the biocatalysts. Different application of different photosynthetic microorganisms often is more suited towards specific types of immobilisation. The application of photosynthetic microorganisms as biocatalysts to produce valuable chemicals or to bioremediate waste streams is often suited towards entrapment within solid materials or behind a membrane. Applications where primary metabolites associated with growth or biomass accumulation is desired, immobilisation via biofilm formation or flocculation is more suitable. This is since entrapment within solid materials often provides higher specific cell loadings and overall higher process efficiencies, but it may restrict microbial growth and may prove challenging to separate the microorganism from the immobilisation material in applications where biomass is the desired product.

Different immobilisation methods are more suited towards different species of photosynthetic microorganisms based on their metabolism, tendency to form biofilms or self-aggregate, and sensitivity to shear, amongst other properties. As a result, the immobilisation method and possible immobilisation material should be selected carefully by taking into consideration the photosynthetic microorganism and application. The different immobilisation methods have shown promise towards efficient and steady PBR performance at laboratory- and pilot-scale, and this is a great advancement towards the end goal of economic and technical feasibility at large-scales.

## Data Availability

The datasets generated during and/or analysed during the current study are available from the corresponding author on reasonable request.
